# A Triumph of Dentistry

**Published:** 1882-04

**Authors:** 


### A TRIUMPH OF DENTISTRY.

At the last meeting of the Medical Society at Strasburg, reported in the *Medical Gazette* of Strasburg, Dr. Julius Böckel presented, in the name of M. Sauval, dentist, a lady for whom the latter had extracted a small molar tooth for dental caries with violent pain ; and, having found it slightly carious to the bottom of its root, he sawed off the points of the root, filled it with gold carefully through the carious channel, and then re-implanted the tooth. The lady was free from all her pain ; the tooth re-established itself solidly in her mouth ; and at the date at which she appeared at the society (three weeks after the operation), the tooth served for mastication as well as her other teeth. This is certainly a remarkable example of what is technically described as dental autoprothesis with aurification.—*Brit. Med. Jour.*

